# Continuous Extrusion Forming Technology of Magnesium Alloy Thin-Walled Tubules

**DOI:** 10.3390/ma16175803

**Published:** 2023-08-24

**Authors:** Xi Yang, Shihan Sun, Zheng Zhou, Xuewen Chen, Guoqing Chen

**Affiliations:** 1 School of Materials Science and Engineering, Henan University of Science and Technology, Luoyang 471023, China; 2 Collaborative Innovation Center of Nonferrous Metals of Henan Province, Luoyang 471023, China; 3 School of Materials Science and Engineering, Dalian University of Technology, Dalian 116024, China; gqchen@dlut.edu.cn

**Keywords:** magnesium alloy, thin-walled tubule, continuous forming, extrusion mold, superimposed billet extrusion method

## Abstract

This paper proposes a new technology of superimposed billet extrusion-forming for thin-walled magnesium alloy tubes. This process represents an improvement over the current technology, which suffers from low production efficiency, poor forming accuracy, and low material utilization. We developed a detailed forming process and mold structure, in which the excess material of the front billet is extruded out of the mold as the rear billet pushes on the front one. Through continuous extrusion, online direct water cooling, and cutting, the automated continuous production of thin-walled tubules is achieved. The optimization of the mandrel structure and its hovering action is also included, with the aim of improving the lifespan of the mandrel and the accuracy of tube size. The numerical simulation method evaluates the effect of the die angle (*α*) on the tube, formed using FORGE NXT 1.1. The results show that for an angle of less than 70°, the defect length of the tube decreases as the die angle decreases, forming an ordered flow of superimposed billets. If the angle is less than 50°, the two adjacently formed tubes separate automatically, with no need for the subsequent cutting process. The best choice of die angle is about 50°, which takes into account the effect of the change in extrusion force.

## 1. Introduction

The coronary artery stent is considered to be one of the most promising technologies for the treatment of coronary artery disease [1]. As a new generation of biodegradable vascular scaffold materials, magnesium alloys have good mechanical properties, with characteristics of biodegradability, and biocompatibility [2,3,4], resulting in broad clinical applications prospects [5,6]. Researchers across the globe have conducted extensive research on magnesium alloy vascular stents and have achieved promising results [5,7,8].

The magnesium alloy vascular stent has an outer diameter of 0.8~2 mm, a wall thickness of 0.03~0.6 mm, and a length of 8~20 mm. It is cut by laser precision cutting from microtubules of the same specifications into a mesh structure that can shrink, expand, and deform. This mesh structure can be deformed by laser cutting from a capillary of the same specifications. The width of the reticulated beam is only 20~150 μm. The diameter of the stent after expansion can reach 6~8 times that before expansion [9,10]. To prevent cracking and fatigue damage during use, the microtubule structure is required to have uniform and fine equiaxed grains, with a grain size not less than 7.6 grade [11,12]. Moreover, the finer the grain, the better the mechanical properties. Research has shown that fine grains are beneficial for improving the corrosion resistance of magnesium alloys [5,13].

The requirements for the material structure and performance of magnesium alloy stents are very strict and can only be satisfied by significant plastic deformation. However, due to the poor plasticity of magnesium alloys, it is difficult to achieve significant plastic deformation through a single deformation method [14].

Many studies have suggested that qualified microtubules can be obtained by preparing thin-walled tubules with larger dimensions through an appropriate extrusion process and by undergoing multiple rounds of rolling, drawing, and heat treatment [10,14,15]. Wang [16] prepared extruded billets with excellent properties through solution treatment and subsequently produced thin-walled tubules for vascular stents by extrusion. In addition, the extrusion billets with ultra-fine-grain structures were obtained through repeated extrusion processes, and thin-walled tubules were obtained after further extrusion [13].

The cases mentioned above indicate that there are different preparation processes for thin-walled tubules, but extrusion molding is the most critical step. Many studies have shown that dynamic recrystallization during extrusion can greatly improve the microstructure of the alloy, thus improving the strength and corrosion resistance of magnesium alloys [16,17,18,19], with an effect that is more obvious than alloying [20]. As for extrusion forming, it is very difficult to continuously form and realize industrial production because the tube diameter is small, the wall thickness is thin and uniform, the subsequent drawing or rolling forming processes require the tube length to be large, the structure of the product is uniform, and the grain size is refined.

Researchers have studied the extrusion-forming method of magnesium alloy thin-walled tubules, and the influence of process parameters on the microstructure and the properties of the products have been discussed. Ge et al. [21] conducted hot compression tests and extrusion trials on AZ31B and ZM21 magnesium alloy tubes. The influence of extrusion parameters on the microstructure and properties of tubes was mainly studied. Through the laboratory hot extrusion system, tubes with an outer diameter of 4~8 mm and an inner diameter of 3~4 mm were formed at 410 °C and a strain rate of 2.78 × 10^−3^/s. The grain size of the two magnesium alloy tubes was 15.3 μm and 14.4 μm, respectively. During the forming process, the billet placed in the mold was formed by extrusion after 10 min of heat preservation. The process belongs to intermittent extrusion. Ge et al. [22] prepared billets with uniform equiaxed grain structure of submicron grain size by multi-pass equal-channel angular pressing (ECAP), and then extruded submicron grain size ZM21 alloy tubules with an outer diameter of 4 mm and an inner diameter of 2 mm at 150 °C at an extrusion ratio of 8. Vedani et al. [23] investigated the relationship between the microstructure, texture, and mechanical behavior of ZM21 and AZ31B magnesium alloy tubes obtained in Reference [21] and provided information for stent design and modeling. Lu et al. [24] prepared JDBM alloy seamless tubules through double extrusion. At a temperature of 350 °C, the solution-treated JDBM billets were first extruded into φ 15 bars with an extrusion ratio of 36, and then tubules were obtained by a second extrusion at a ratio of 57, with an outer diameter of 3.5 mm, a wall thickness of 0.25 mm, and a grain size of 2 μm. Li et al. [25] prepared Mg-2Zn-0.46Y-0.5Nd thin-walled extruded tubes with an outer diameter of 43.0 mm, a wall thickness of 0.32 mm, and an average grain size of 8.6 μm, using the water-cooled large extrusion ratio method. The extrusion ratio was 112, and the billet was machined from solution-treated and extruded bars. Kou et al. [26] used solution-treated ZE21B machined billets and extruded them at 380 °C, an extrusion speed of about 6 mm/s, and an extrusion ratio of 105, and obtained tubes with an outer diameter of 3.6 mm, an inner diameter of 3.05 mm, and a grain size of 4.66 μm. Duan et al. [27] used a fixed mold with a heating device to extrude a magnesium alloy tube with an outer diameter of 20 mm and a wall thickness of 0.6 mm, reducing labor intensity and improving efficiency. However, similar to Reference [17], the same problem of low material utilization and productivity existed.

The above studies have provided beneficial discussions on the effects of billet treatment, mold structure (die angle, inlet fillet, working belt length, etc.), and process conditions (forming temperature, extrusion speed, extrusion ratio) on the extrusion-forming process and product structure and properties. However, these studies are still in the experimental stage, using simple molds and manual operation, resulting in high labor intensity and low production efficiency. The tube is bent heavily, with uneven wall thickness, and the short length of the tube affects the subsequent deformation process. There have been no reports on high-efficiency and high-quality continuous production techniques for magnesium alloy thin-walled tubules. 

This study addresses the issues present in the current forming processes. We discuss optimizing the mold structure, stabilizing the forming quality, facilitating continuous production, and increasing the length of the tube. A design is proposed for a high-quality, automated, continuous production magnesium alloy thin-walled tubule extrusion forming device.

## 2. Overall Design Idea

**Figure 1:**
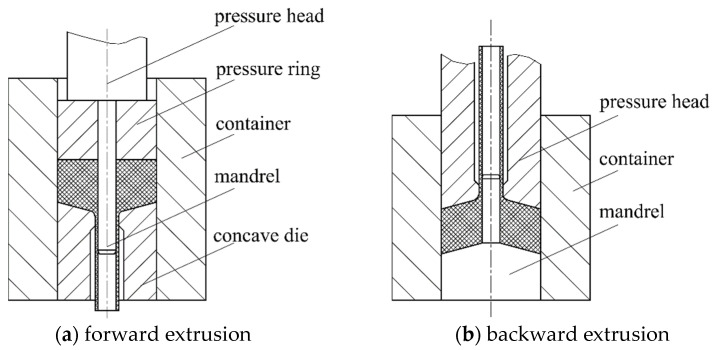
Schematic diagram of a conventional extrusion die.

The existing magnesium alloy thin-walled tube extrusion process adopts conventional forward and backward extrusion-forming processes; the working principle diagram of the die is shown in Figure 1. In this process, the forward extrusion pressure head and the mandrel are integral or combined structures, with a synchronous moving. When forming, the pressure head drives the pressure ring downward to press the tube billet. After completion, the pressure head and the mandrel return, and the formed thin-walled tubule and the pressure ring rise together as a result of the clamping force. After the thin-walled tubule exits the container, a stripper plate (not shown) installed on the container prevents the pressure ring from continuing upward, and the tube is removed from the mandrel. The mandrel of the backward extrusion die is fixed at the lower part of the container. When the pressing head returns, the tube stays on the mandrel, then it is pulled out and demolded manually.

Conventional forming processes and dies can lead to some serious defects due to the unique structure of the thin-walled tubules. (1) The clad length of the tube on the mandrel increases with the extrusion process, resulting in an increase in friction and forming pressure. (2) There are process residues. (3) It is difficult to form a long tube due to the restrictions associated with the equipment operating space. (4) The forward extrusion die can only use a mandrel with an equal diameter, which is small in diameter, long in length, subjected to large tensile stress, and easy to break; the deformation resistance of the backward extrusion process is small, but demolding is very difficult. (5) The process involves low production efficiency and high labor intensity.

Aiming at solving the existing problems in the current forming process, comprehensive consideration was given to three aspects: the forming equipment, the forming process, and the forming die, to coordinate the design and make them form an organic whole. By adopting the superimposed tube billet extrusion method, the rear billet is used to push the front billet. The tube is completely formed and extruded from under the die, and then the tube is straightened and cut online, thus realizing the continuous extrusion process. Independent mandrel and matched stroke coordination devices are adopted to suspend the mandrel in a fixed position in the concave die, which does not move with the downward movement of the pressure head, thereby improving the precision of the extruded tubes. At the same time, the service life of the mandrel is prolonged by optimizing the mandrel structure.

## 3. Forming Technology Scheme

### 3.1. Selection of Forming Equipment

(1) Equipment type selection

The extrusion formation of thin-walled tubes requires stable pressure and speed. The two types of presses typically used for extrusion molding are a crank press and a hydraulic press. The nominal pressure stroke produced by the crank press is short, and it is difficult to adjust the working speed to enable downward pressure at a uniform speed. A crank press with a servo motor can solve this problem, but the equipment structure is complex and the cost is high. A hydraulic press can realize this function and has a simpler structure and lower cost. Thus, the hydraulic press is preferred.

Hydraulic presses are divided into vertical and horizontal types. Metal tubes with larger diameters generally use a horizontal hydraulic press, as the formed tube is longer, and it is more convenient to cut into sections and remove. By contrast, the extrusion of small-diameter magnesium alloy tubes should adopt a vertical hydraulic press. The mold has high centering precision, the billet temperature and craft lubricant are evenly distributed, and the parallelism of the movable parts of the press is high. Thus, the wall thickness difference of the tube is small, and longitudinal bending will not occur due to gravity. Tubular products do not require a lower ejection device, but there should be enough space at the lower part of the press to facilitate the removal of the tubes.

(2) Calculation of extrusion force

In the design of an extrusion die, the products and extrusion equipment should be comprehensively considered and the die parameters should be reasonably selected to ensure that the extrusion equipment can provide sufficient extrusion force.

When designing an extrusion die, the product and the extrusion equipment should be considered, and the die parameters reasonably selected to ensure that the extrusion equipment can provide enough extrusion force. Extrusion force is mainly related to extrusion process parameters strength, state, and length of billet materials. The calculation of extrusion force *F* is based on the empirical Equation (1):

*F* = *p*π(*D*_t_^2^ − *d*_t_^2^)/4 (1)

(2) $$p=\sigma_{s}\left[\left(1+\frac{1}{\sqrt{3}}\cot\alpha \frac{\bar{D}+d_{1}}{\bar{D}}\right)\ln\lambda +\frac{4\;\mu L}{D_{1}-d_{1}}+\frac{4}{\sqrt{3}}\times \frac{h}{D_{t}-d_{t}}\right]$$

where *p* is extrusion pressure (MPa), and is calculated by Equation (2); *D*_t_ is the inner diameter of concave die (mm); *d*_t_ is the diameter of the mandrel reinforcement section (mm); *σ*_s_ is the yield strength of deformed metal (MPa); *α* is the die angle of concave die; *λ* is the extrusion ratio, that is, the ratio of the cross-sectional area of billet to the cross-sectional area of product; and *μ* is the friction coefficient. For warm extrusion, when the lubrication effect is good, it is advisable to set *μ* at 0.2~0.25. $\bar{D}$ is the average diameter of the billet in the plastic deformation zone (mm), that is, $\bar{D}$ = 0.5(*D*_t_ + *D*_1_); *D*_1_ and *d*_1_ are the diameter of the concave die orifice and thin-walled tubes, respectively (mm); and *h* is the height of the billet in the undeformed zone (mm), which can be approximately estimated by the original height of the extruded billet [28].

### 3.2. Billet Shape and Size

(1) Size of extruded tube

The inner and outer diameters of the extruded thin-walled tubule are determined based on the subsequent process. To facilitate the subsequent rolling and drawing process, the length should be as long as possible under the conditions allowed by the equipment; ideally, more than 1000 mm.

(2) Billet shape

Extrusion billets can be solid or hollow. Shunt welding extrusion and piercing extrusion are the two extrusion methods used for solid billets. The former does not meet the use requirements of thin-walled tubes for vascular stent. The latter cannot guarantee the coaxiality requirements between the inner hole and the outer circle of the billet, and is unable to carry out continuous production due to the surplus material that is generated during the piercing process and because the piercing needle is prone to breaking. In addition, the support has very high requirements for the size, coaxiality, surface quality, and mechanical properties of the thin-walled tube, so it is necessary to use the finishing tube billets with high coaxiality.

(3) Billet size

According to the structure and fixing mode of the die mandrel, set *d* ≥ *d*_1_, and *D* is determined by Equation (3):

*D* = [*λ*(*D*_1_^2^ − *d*_1_^2^) + *d*^2^]^1/2^ (3)

where *D* and *d* are the outer and inner diameters of the billet (mm), respectively.

The billet length is calculated according to the equal volume principle:

*H* = *L*(*D*_1_^2^ − *d*_1_^2^)/(*D*^2^ − *d*^2^) (4)

where *H* is billet height (mm) and *L* is extruded tube length (mm).

### 3.3. Forming Process Requirements

#### 3.3.1. Extrusion Ratio

To improve the grain-refining effect, a larger extrusion ratio, λ, should be selected. However, with an increase in λ, the extrusion force will also increase, and the die will fail. For the extrusion forming of thin-walled tubes, λ = 80~120.

#### 3.3.2. Extrusion Forming Temperature

The temperature of tube extrusion is related to the material used to form the tubes; generally between 350 °C and 450 °C. If the temperature is too high, it will lead to grain growth and corrosion oxidation. If the temperature is too low, it will reduce the plasticity and increase the resistance of the material, which is not conducive to forming. Specifically, the tubes should be formed at a temperature that is below the recrystallization temperature of the material, to form a large deformation grain structure. Then the grain can be refined through recrystallization treatment. Dynamic recrystallization can also be used for superplastic forming to obtain a fine-grained structure. The mold temperature can be equal to or slightly lower than the billet temperature to form isothermal deformation conditions and improve the plastic deformation ability of the billet.

#### 3.3.3. Extrusion Deformation Speed

Extrusion deformation velocity refers to the pressing velocity of the pressing head. The higher the value, the higher the production efficiency. The pressing velocity of the pressing head has a direct impact on the material flow velocity in the deformation zone. During the forming process, the material in the deformation zone near the working zone of the die flows at a high-speed plasticity. When passing through the working zone, the flow rate is equal to λ times the pressing velocity. Heat is generated by plastic deformation energy and friction with the material and the mold cavity/mandrel so that the temperature of the material in the deformation zone rises. When the temperature exceeds a certain value, it will cause grain growth and surface quality degradation of the tube, thus limiting further improvement on deformation velocity.

#### 3.3.4. Lubricants

There are many kinds of lubricants suitable for magnesium alloy extrusion forming, and oil-based graphite is more commonly used in the existing literature. However, during mass production, a large amount of pollution is generated. Therefore, this study recommends using graphite emulsion, which emits less pollution into the atmosphere and can be sprayed, brushed, or dipped. Spraying will produce dust, which requires closed operation, and brush coating is not uniform and it is not easy to coat the inner hole of the billet; however, dip-coating is convenient and suitable for automatic operation. After the dirt of the billet is removed, it is heated to 160~200 °C, immersed in aqueous graphite, stirred quickly, lifted and drained or dried for later use. The above factors not only affect the forming force but also impact the material temperature in the deformation zone, which in turn influences the microstructure of the tube. In order to achieve the appropriate grain refinement, it is not only necessary to design a high extrusion ratio but also to control the material temperature in the deformation zone. After the lubricant is selected, the billet temperature, the die temperature, and the extrusion speed will affect the material temperature in the deformation zone. Therefore, it is important to coordinate these three factors. For instance, when the billet temperature and the die temperature are low, the deformation speed can be appropriately increased; otherwise, the deformation speed should be reduced. In short, the temperature in the deformation zone should not be higher than the recrystallization temperature so as to avoid coarse grain structure. See Table 1 for extrusion-forming conditions and performance characteristics of different magnesium alloy thin-walled tubules.

### 3.4. Structure and Working Principle of Extrusion Shaping Dies for Superimposed Tube Billet

**Figure 2:**
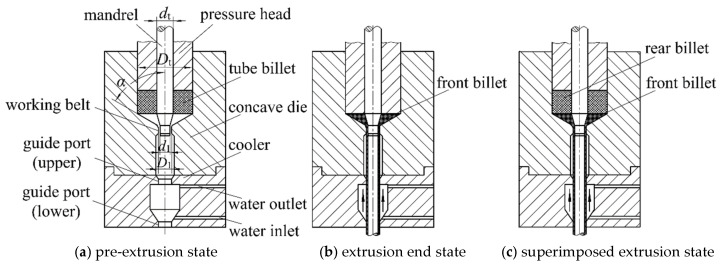
Schematic diagram of extrusion forming of superimposed tube billet.

The die structure principle of thin-walled tubule superimposed tube billet extrusion forming is shown in Figure 2. The technological process is as follows. The tube billet is covered with a lubricant, heated to the forming temperature, and put into the preheated mold cavity, as shown in Figure 2a. The mandrel and the pressure head descend; the mandrel descends to the set position to keep still and the pressure head descends under pressure so that the tube billet generates plastic flow, which is extruded from the annular gap formed by the working belt of the female die and the mandrel. The tube billet becomes a seamless thin-walled tube that is straightened by the straightening belt, and the tail of the tube billet is left with frustum residue, as shown in Figure 2b. The mandrel and the pressure head return to their initial positions, and then the tube billet is put in again, as shown in Figure 2c, for superimposed and extrusion forming. Under pressure from the rear tube billet, the frustum leftover from the front tube billet continues to form. The rear tube billet then enters the deformation zone to produce plastic deformation. The extruded tube is subjected to on-line cooling through a water cooler directly connected to the mold to ensure a fine-grained structure. The two guide ports within the cooler can also function as straightening belts, performing secondary straightening on the tube and ensuring its straightness. By cutting horizontally from the joint position of adjacent tubes, the long thin-walled tubules can be obtained.

**Figure 3:**
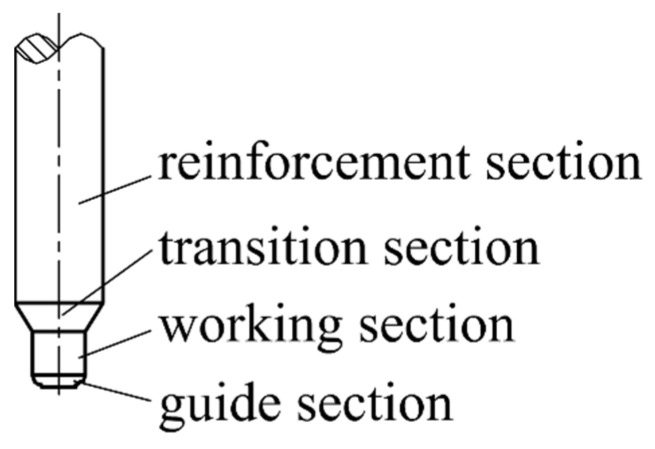
Mandrel.

**Figure 4:**
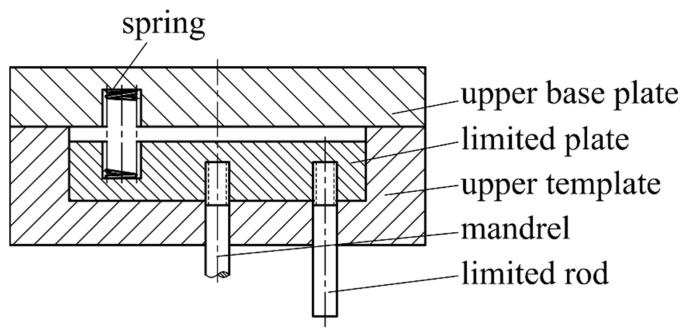
Schematic diagram of a coordinated extrusion die holder.

The key points of die structure design are as follows:

(1) To ensure the uniform wall thickness of the tube, the mold’s squeeze cylinder, the working belt of the die, and the forming end of the mandrel should have high coaxiality. Among these factors, the coaxiality between the container and the working belt of the cavity die can be guaranteed by the precision of the cavity die. However, there are many dimensional chain links between the mandrel and cavity die. In addition to ensuring that all related die parts have small form and position tolerance, the upper and lower dies must be guided accurately. It is recommended to adopt the rolling guide post-guide mechanism. In addition, the coaxiality requirement of the inner and outer circles of the tube billet and the uniformity of the structure should be ensured, both of which will affect the coaxiality of the tube billet in the way of error reflection.

(2) Thin-walled tubes are small in diameter and wall thickness, and due to the influence of metal deformation, uneven flow, and friction, the extruded thin-walled tubes are easy to bend, which adversely affects subsequent processes. Therefore, a correction ring can be arranged below the working belt to directly straighten the extruded thin-walled tubes. The diameter of the correction ring is the same as that of the working belt, and the distance between the correction ring and the working belt is about 4~6 times of the diameter of the tube. There is a high requirement for coaxiality between the correction ring and the working belt.

(3) Adopt the optimized reinforced mandrel structure, as shown in Figure 3. Thickening the mandrel at the tube billet to form a reinforced section can effectively increase the overall strength and stiffness of the mandrel. The part corresponding to the working belt of the female die is the working section of the mandrel, and its length is short, which can effectively reduce the wrapping force of the tube. The cone-platform transition section can generate reverse thrust to the mandrel, offset some friction, and optimize the stress environment of the mandrel.

(4) When pressing, the mandrel stops moving after descending to a predetermined height, and the pressure head continues to descend for extrusion forming. The double-acting hydraulic press can be programmed to conveniently control the pressure head and mandrel to complete the given action. When the press does not have double-acting conditions, the coordinated die holder as shown in Figure 4 can be used.

The upper base plate of the die base is used for fixing the pressure head, and a limited plate capable of axial movement is also installed in it. The limited plate is fixed with the mandrel and the limited rod and is at the lower starting point under the action of the spring.

When the press goes down, the limited rod touches the lower die part to stop moving, and the mandrel stays at a predetermined position through the limited plate. The press continues to descend, and the upper base plate drives the pressure head to descend for extrusion forming. When returning, the press drives the upper base plate and the pressure head upward for a certain distance and then drives the mandrel to return.

(5) Use a direct water-cooling device, as shown in Figure 2. This device has two guide ports: one upper and one lower. When the tube passes through, it can seal the water channel and activate the external water recirculation system. The cooling water is drawn into the water channel from bottom to top, forming a negative pressure flow. This prevents the leakage of the cooling water. The cooling water flow and tube movement travel in opposite directions, which can enhance the cooling effect.

## 4. Concave Die Angle Design

There is a bonding issue between the two continuous extrusion forming tubes, while they are stacked billets. The defect areas (including the assembled part and the incomplete section) at the joint of the two tubes must be cut off, based on the quality requirements of thin-walled thin tubes. The size of the die angle of the concave die not only affects the forming force but also the length of the defect area, which then affects the tube forming quality and material utilization rate. Here, we evaluate the effects of concave die angle for the tube forming result and forming forces and obtain the best angle using a numerical simulation method.

### 4.1. Simulation Condition

The forming billet is a Φ25 mm × Φ6 mm × 8 mm tube coated with water-based graphite lubricant, and the billet material is AZ31B. The superimposed extrusion process is used to form a thin-walled tube with the size of Φ4 mm × Φ3.2 mm. The extrusion ratio is 100 to obtain a fine-grained structure. The cone angle of the mandrel is 30°, and the working belt length of the concave die is 2 mm. The die angle is selected between 40° and 90°. In this way, the die structure is determined. Figure 2a shows the diagram of the die structure with the angle at 60°.

**Figure 5:**
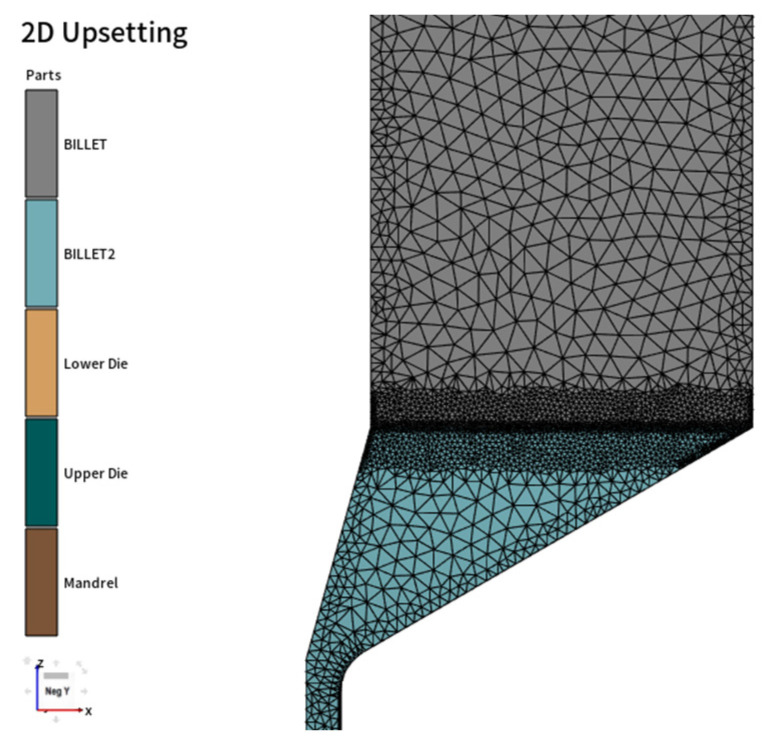
Finite element mesh model.

The extrusion forming of the magnesium alloy thin-walled tubule is numerically simulated by FORGE-2D, using a viscoplastic finite element model. During the simulation process, the deformation of the mold is not considered, and it is set as a rigid body. The lubrication is good between the front and rear billets, as well as between the billets and the mold, and the Coulomb friction model is chosen as the friction model. When the front billet is pressed down to a certain position (the pressure head moves to the top of the transition section of the mandrel or the conical surface of the concave die), put in the rear billet and divide the grid, as well as re-divide the grid of the deformed front billet. The two billets are automatically divided using tetrahedral grids. The parameters set for the numerical simulation of superimposed billet continuous extrusion forming are shown in Table 2. Considering the symmetry of the tubes and molds, a 1/2 model is adopted to improve computational efficiency. The finite element model is shown in Figure 5.

### 4.2. Analysis of Simulation Results

**Figure 6:**
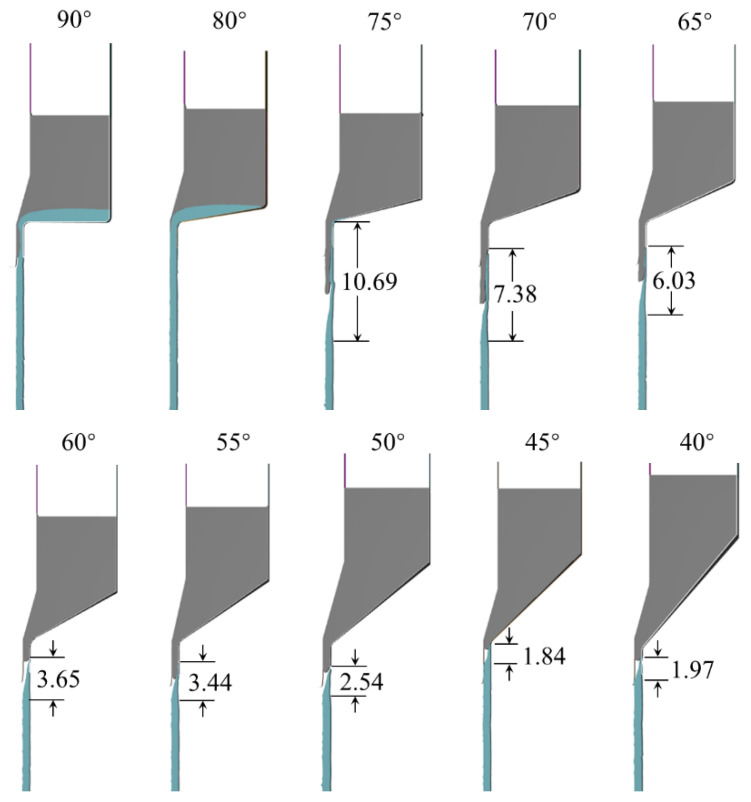
Tube defects with different concave die angles (unit: mm).

Figure 6 shows the simulation result of billet extrusion forming tubes at different concave die entry angles. It shows that there is still a large amount of surplus material that stays in the concave die at the front tube billet when the rear tube billet begins to squeeze into the die in the case of die angles 90° and 80°. The subsequent extrusion leads to the surplus materials wrapping around the tube formed by the rear tube billet. This forms a long-distance layered coating tube that becomes a waste product, and deformation dead zones appear. Therefore, forming the tube at die angles from 80° to 90° is not suitable.

**Figure 7:**
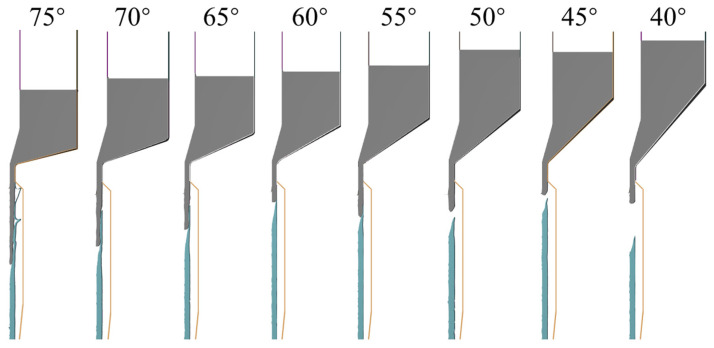
Combination of formed tubes.

When the die angle is 75°, the front tube billet can be smoothly extruded into a tube, but the wrapping length at the junction is long. Furthermore, the outer layer exhibits folding instability, making it difficult to form stably. For die angles less than 70°, the length of defects with incomplete coverage and cross-section varies with the angle decrease, as shown in Table 3. This is because it becomes easier for the outer metal to flow towards the die hole at a small die angle, forming an extrusion forming process where the superimposed billet flows in an orderly manner. Figure 7 shows the situation of tube separation, where the formed tube can automatically fall off the tube above when the connection area descends below the mold hole working zone, once the die angle is less than 50°.

**Figure 8:**
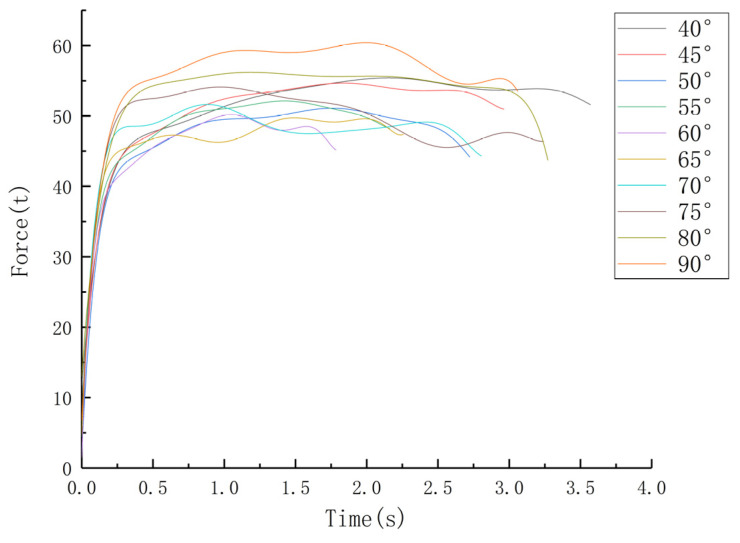
Extrusion force curve during superimposed extrusion process.

Figure 8 shows that the extrusion forces vary with the die angle, and Table 3 shows the maximum extrusion force. The extrusion force is almost the same when the die angle is between 50° and 70°, and increases significantly once the angle is 45°.

In summary, the defect length of the tube decreases as the die angle decreases, and for angles less than 70°, superimposed billet extrusion forming is a suitable technological process. Moreover, the two adjacent formed tubes separate and the front completely extruded tube falls automatically at angles less than 50°. Thus, there is no need for the online cutting process, making the tube-forming process simpler. As a result, the best choice of die angle is about 50°, taking into account the effect of extrusion force.

## 5. Results and Discussion

Currently, the main concern stemming from research into magnesium alloy vascular stents is the quality and clinical application. Concerning thin-walled tubules, most of the research focuses on the impact of the forming process parameters on the quality of tube forming. The goal is to form tubes with small grain sizes that meet performance requirements, yet forming efficiency and price issues have been ignored. Most studies use simple movable type molds [13,14,15,29]. The molds are put into the furnace to heat and hold for 2~2.5 h, then moved to the press table for extrusion, and finally, the tube is taken out after disassembling the molds. Therefore, it is not possible to produce continuously. The forming cycle of a single tube is very long, the quality is difficult to control, and the cost is very high. There are also fixed molds that can be heated in real time [21,27], with a significant reduction in operating time and labor. However, as with the movable type mold, it is also an intermittent extrusion mode, which always generates residual extrusion waste. The tube with residual waste needs to be taken before being extruded again. Demolding is difficult, material utilization is low, and the formed tube is relatively short due to the limitations of equipment operating space and ejection stroke, which affects the subsequent rolling and drawing processes.

This study aimed to enhance production efficiency and cut costs by utilizing existing, mature forming methods. With a focus on maintaining quality, we aimed to improve the technology and develop an automated forming mold. Specific improvements involve continuous extrusion forming, achieved by superimposed billets; setting a direct water-cooling and straightening composite device to ensure fine grain and straightness of the tube; installing a spindle-hovering mechanism to stabilize the section size of the tube; strengthening the mandrel structurally to extend its lifespan; optimizing the die angle, reducing the length of the joining area of adjacent tubes, and discovering the proper die angle at which the extruded tube will automatically fall, eliminating the need for cutting the tube. Compared to the extrusion-forming methods of thin-walled and small-diameter tubes in the existing literature, the advantages of this research are detailed as follows:

(1) The existing forming methods have the disadvantages of difficulties involved with demolding, large labor requirements, low material utilization, and short length of the workpiece, resulting in high costs. This study proposes a superimposed billet extrusion method, using a rear tube billet to drive a front tube billet to deform and extrude the remaining material into a tube. This solution allows for continuous production, easy demolding of parts, high production efficiency and material utilization, and the ability to form longer tubes.

(2) In the existing forming method, the mandrel gradually penetrates into the tube during extrusion, so its diameter can only be as large as the inner diameter of the tube, which may be less than 3 mm, while the length can be up to 20 mm or more. Due to the friction between various parts, the working conditions are harsh and the mandrel is easily broken. To improve the service life, the mandrel can be equipped with a smaller demold slope, but this will result in inconsistencies in the inner diameter of the tube. In this study, the hovering mandrel is set, and its working section is fixed, which reduces the flow resistance and improves the consistency of the tube size. Moreover, the reinforced structure can be used to optimize the stress environment and significantly improve the service life.

(3) The numerical simulation results indicate that the appropriate selection of die angles not only enables the formation of an orderly extrusion-forming process with superimposed billet flow, reducing the length of the connection area between adjacent tubes, but also enables the automatic separation and release of formed tubes, thereby eliminating the need for subsequent cutting processes.

(4) Based on an extrusion speed of 1 mm/s, this study estimates that it takes approximately 10~15 s to form an 800 mm tube. Although the mold cost of this research technology is relatively high, considering mass production, the cost of the mold can be negligible when converted to each tube, and considering the time cost, its economic benefits are much higher.

One important future direction of the study is the development and test of the forming mold, as well as the microstructure and performance testing of the products, to produce qualified tubes, realize the automatic heating and feeding of billets, and ultimately realize the industrial production of thin-walled tubules.

## 6. Conclusions

This paper aims to improve the low production efficiency, high labor intensity, poor product precision, and short die life for the extrusion forming of thin-walled magnesium alloy tubes, and proposes a novel technology with the design of an extrusion-forming device capable of automatic, continuous production that optimizes the forming process and die structure.

(1) We propose a forming scheme in which the rear tube billet pushes the front tube billet to form. All leftover materials are formed into tubes, thus realizing continuous production, convenient demolding, high production efficiency, and material utilization rate.

(2) The proposed reinforced hoving mandrel optimizes the structure and improves the stress distribution, bearing capacity, and service life.

(3) There is a fixed sizing belt between the mandrel and the female die, which keeps the deformation condition of the billet deformation zone unchanged during the whole forming process. Thus, the forming force is small and the size has high consistency.

(4) We designed a composite device of direct water cooling and straightening to ensure the fine grain and straightness of the tube.

(5) Through numerical simulation, we determined that the optimal die angle is about 50°, with low forming force and small defects at the joint of the tubes. The formed tubes can automatically separate and fall.

## Figures and Tables

**Table 1:** Extrusion process parameters and microstructure performance of different magnesium alloy thin-walled tubules.

Billet Material	JDBM [13]	WE43 [15]	ZE21B [26]	AZ31 [27]	AZ61 [29]
Billet state	Solution treatment + squeeze	Solution treatment	Solution treatment	Solution treatment	Solution treatment
Diameter and wall thickness of tube/mm	φ3.5 × 0.25	φ10 × 1.5	φ3.6 × 0.275	φ20 × 0.6	φ8 × 1
Press	Horizontal	Vertical	Vertical	Vertical	Vertical
Extrusion ratio	57	69.6	105	35	43
Billet temperature/°C	380	450	380	400	400
Mold temperature/°C	380	430	380	380	300
Lubricant	Boron nitride	Oil-based graphite	Graphite emulsion	Oil-based graphite	Oil-based graphite
Extrusion speed/(mm/s)	1	2	6	0.1	2
Force/kN	-	1700	-	1320	-
Tensile strength/MPa	267.5	-	-	286	245.6
Yield strength/MPa	220	-	-	-	202.3
Extensibility/%	48.8	16.6	-	15.6	6.7
Grain size/μm	2	5.7	4.66	-	-

**Table 2:** Setting parameters for extrusion process simulation.

Project	Value
Initial temperature of billet/°C	400
Initial temperature of Die/°C	400
Extrusion speed/(mm·s^−1^)	1
Extrusion ratio	100
Coulomb friction coefficient	0.2
Default mesh size/mm	0.4641
Refined mesh size/mm	0.1
Fine front factor	2
Heat transfer coefficient/(1000 W/m^2^·K)	10,000
Height storage step/mm	0.085

**Table 3:** Defect length and maximum extrusion force of tubes with different die angles.

*α*/°	40	45	50	55	60	65	70
Defect length/mm	1.9	1.84	2.54	3.44	3.65	6.03	7.38
Maximum extrusion force/t	55.4	54.66	51.1	52.1	50.18	49.74	51.62

## Data Availability

Not applicable.
